# Dynamic metabolic profiling together with transcription analysis reveals salinity-induced starch-to-lipid biosynthesis in alga *Chlamydomonas* sp. JSC4

**DOI:** 10.1038/srep45471

**Published:** 2017-04-04

**Authors:** Shih-Hsin Ho, Akihito Nakanishi, Yuichi Kato, Hiroaki Yamasaki, Jo-Shu Chang, Naomi Misawa, Yuu Hirose, Jun Minagawa, Tomohisa Hasunuma, Akihiko Kondo

**Affiliations:** 1 State Key Laboratory of Urban Water Resource and Environment, School of Municipal and Environmental Engineering, Harbin Institute of Technology, Harbin 150090, P.R. China; 2 Graduate School of Science, Technology and Innovation, Kobe University, 1-1 Rokkodai, Nada-ku, Kobe 657-8501, Japan; 3 Division of Environmental Photobiology, National Institute for Basic Biology, Okazaki 444-8585, Japan; 4 Department of Chemical Engineering, National Cheng Kung University, Tainan 701, Taiwan; 5 Research Center for Energy Technology and Strategy, National Cheng Kung University, Tainan 701, Taiwan; 6 Department of Environmental and Life Science/Electronics-Inspired Interdisciplinary Research Institute (EIIRIS), Toyohashi University of Technology, 1-1 Hibarigaoka, Tempaku, Toyohashi, Aichi 441-8580, Japan; 7 Department of Basic Biology, School of Life Science, SOKENDAI (The Graduate University for Advanced Studies), Okazaki 444-8585, Japan; 8 Department of Chemical Science and Engineering, Graduate School of Engineering, Kobe University, 1-1 Rokkodai, Nada-ku, Kobe 657-8501, Japan; 9 Biomass Engineering Program, RIKEN, 1-7-22 Suehiro-cho, Tsurumi-ku, Yokohama, Kanagawa 230-0045, Japan

## Abstract

Biodiesel production using microalgae would play a pivotal role in satisfying future global energy demands. Understanding of lipid metabolism in microalgae is important to isolate oleaginous strain capable of overproducing lipids. It has been reported that reducing starch biosynthesis can enhance lipid accumulation. However, the metabolic mechanism controlling carbon partitioning from starch to lipids in microalgae remains unclear, thus complicating the genetic engineering of algal strains. We here used “dynamic” metabolic profiling and essential transcription analysis of the oleaginous green alga *Chlamydomonas* sp. JSC4 for the first time to demonstrate the switching mechanisms from starch to lipid synthesis using salinity as a regulator, and identified the metabolic rate-limiting step for enhancing lipid accumulation (*e.g.*, pyruvate-to-acetyl-CoA). These results, showing salinity-induced starch-to-lipid biosynthesis, will help increase our understanding of dynamic carbon partitioning in oleaginous microalgae. Moreover, we successfully determined the changes of several key lipid-synthesis-related genes (*e.g.*, acetyl-CoA carboxylase, pyruvate decarboxylase, acetaldehyde dehydrogenase, acetyl-CoA synthetase and pyruvate ferredoxin oxidoreductase) and starch-degradation related genes (*e.g.*, starch phosphorylases), which could provide a breakthrough in the marine microalgal production of biodiesel.

Increasing global energy demands have resulted in considerable effort to construct a recycling-oriented society utilizing novel sustainable energy sources^1^. Biomass has significant potential as an effective, environmentally benign renewable energy source that is biodegradable and non-toxic^2^. Microalgae are promising biodiesel producers due to their high growth rate, high CO_2_ fixation efficiency, high environmental stress tolerance, and the capacity to accumulate a large amount of lipid without competing for arable land and potable water^2^,^3^,^4^,^5^. However, the commercial production of microalgal biodiesel currently faces the serious challenges of low lipid productivity and high production costs^2^,^6^,^7^. Enhancing the economic feasibility of microalgal biodiesel will require critical engineering innovations in large-scale cultivation and breakthroughs for regulating lipid metabolism^8^.

Microalgae typically accumulates energy-rich compounds such as lipid and starch under environmental stress such as nitrogen depletion and high salinity by redirecting carbon flow towards these compounds for storage^6^,^8^,^9^. Synthetic pathway to produce lipid and starch by CO_2_ fixation have been determined in *Chlamydomonas reinhardtii (C. reinhardtii*) (Fig. 1); the genes encoding phosphoglucoisomerase (PGI), phosphoglucomutase (PGM), ADP-glucose phosphorylase (AGPP), α-amylase (AMY) and starch phosphorylase (SP) are related to starch biosynthesis and degradation^10^,^11^. Also, the genes encoding pyruvate dehydrogenase (PDH), pyruvate-ferredoxin oxidoreductase (PFOR), acetyl-CoA (AcCoA) carboxylase (ACC), pyruvate decarboxylase (PDC), acetaldehyde dehydrogenase (ALDH), AcCoA synthetase (ACS), and glycerol 3-phosphate dehydrogenase (GPDH) are related to lipid biosynthesis^11^,^12^,^13^,^14^,^15^,^16^,^17^. The compartmentalization of carbon metabolism in *C. reinhardti* was reviewed by Johnson *et al*. in detail^9^. Siaut *et al*. established that microalgae initially synthesize starch for a short-term energy reserve, then lipid is synthesized as the long-term energy storage compound^18^. Thus, previous studies have demonstrated that the lipid content of microalgae can be enhanced by switching carbon partitioning from starch to lipid synthesis using starch-related-gene-knockout approaches, while the cell growth of such engineered strains is inhibited^18^,^19^,^20^,^21^. Slower cell growth will result in a decreased lipid productivity even if the lipid content is improved.

Another current major limitation of microalgal biodiesel production is the shortage of freshwater resources^22^. This highlights the importance of developing microalgal strains that are highly tolerant of seawater. Moderate salinity stress may induce lipid accumulation in some microalgae, but also may cause growth inhibition^8^,^23^.

Metabolomics is a powerful tool for functional biology^24^,^25^,^26^ and allows the comprehensive profiling of metabolite accumulation in specific biosynthesis pathways^21^. Although several studies have focused on the pool size of key metabolites during partial metabolic profiling, there are no reports describing the regulation of “dynamic” metabolism in microalgae^27^,^28^. In addition, transcriptomic analysis is vital for providing comprehensive profiling of the mRNA levels of key enzymes related to environmental stress^29^,^30^.

We have developed feasible methods for lipid production using the oleaginous green alga *Chlamydomonas* sp. JSC4, which was isolated from the south Taiwan coast^23^,^31^,^32^. As a hopeful biomass producer, JSC4 possesses advantageous characteristics such as high growth potential and high starch/lipid content^23^,^31^. Importantly, lipid accumulation in JSC4 is effectively triggered by addition of sea salt (SS) in combination with nitrogen depletion, and the highest lipid productivity of 312 mg L^−1^ d^−1^ per unit dry cell weight (DCW) was attained previously under the photoautotrophic conditions^31^. Also, dynamic metabolic profiling using an *in vivo*^13^C labeling was carried out under different light intensities to reveal carbon metabolism after CO_2_ fixation^31^,^33^.

For efficient lipid production by microalgae, it is required to understand the regulatory networks that control carbon partition from starch to lipid^9^. However, the starch-to-lipid switching mechanisms in *Chlamydomonas* spp. under the salinity condition has yet to be elucidated. In this study, the switching mechanism was explored by monitoring *in vivo* dynamic metabolic profiles. Furthermore, mRNA regulation of the key genes involved in lipid biosynthesis and starch biosynthesis/biodegradation was assessed for the first time in JSC4. This study demonstrates that salinity is a powerful regulator for switching lipid/starch biosynthesis, and reveals the key mechanisms for maximizing the lipid production in JSC4.

## Results

### Feasibility of using JSC4 as a biodiesel producer

The growth performance of JSC4 under salinity stress was investigated by cultivating under 0%, 1%, or 2% (*w*/*v*) SS (Fig. 2). High biomass levels were obtained in medium containing 0% and 1% SS (5.6 g L^−1^ and 6.4 g L^−1^ after 9 days’ cultivation, respectively). JSC4 exhibits significant lipid accumulation more than 45% of DCW under 1% and 2% SS, which is obviously superior than the lipid content triggered by single stress of nitrogen depletion (Table 1). This result indicates that the salinity stress coupled with nitrogen depletion significantly improves the lipid content of JSC4. The highest lipid productivity of 358 mg L^−1^ d^−1^ was provided under 1% SS, which is superior than our previous results (in the range of 233–312 mg L^−1^ d^−1^)^23^,^31^ and previous studies (Table S1).

### Energy storage compound switched from starch to lipid under salinity stress

Since most of the carbohydrate in *Chlamydomonas* spp. is starch^19^, we compared the time-course profiles of the total carbohydrate and total lipid content under different SS concentrations (Fig. 2 and Table 1). At 0% SS, the carbohydrate content sharply increased from 33.8% (Day 2) to 61.3% (Day 4) and remained constant thereafter, whereas the lipid content gradually increased and attained a maximum value of 32.8% (Day 8). The carbohydrate content in 1% or 2% SS also increased to around 60% during early-stage culture (Day 2 to Day 4), but then dramatically decreased after 4 days’ cultivation, in parallel with an increase in the lipid content. A maximum lipid content of 46.5% and 56.9% was obtained in 1% and 2% SS, respectively. These results illustrated that the dominant energy storage compound was rapidly switched from starch to lipid upon salinity stress. In addition, we observed salinity-induced lipid accumulation under a transmission electron microscope (TEM). The images depicted in Fig. S1 clearly show many large oil drops formed under salinity stress. Taken together, we demonstrate that energy-flow was shifted from carbohydrate (mainly starch) to lipid by salinity stress.

### Time-course profiles of key metabolite in starch and lipid synthesis with/without salinity stress

To investigate the metabolic mechanism at the lipid production stage, JSC4 cells were cultivated under 0% or 2% SS for 3, 5 and 7 days, and the intracellular metabolites were analyzed comprehensively. In the whole metabolites, we focused on the key metabolites related to starch and lipid synthesis and quantified the pool sizes (Fig. 3). The pool size of 3-phosphoglycerate (3PGA) was similar under the two conditions. Starch is synthesized in *Chlamydomonas* spp. cells by the polymerization of ADP-glucose (ADP-Glc)^19^. The pool sizes of the sugar phosphates (*i.e*., fructose 6-phosphate (F6P) and glucose 6-phosphate (G6P)) were significantly higher under 0% SS at Day 5 and 7. Taken these results with the carbohydrate content shown in Fig. 2, high pool sizes of these metabolites would be corresponding to high carbohydrate content under 0% SS.

Lipid is biosynthesized from glycerol 3-phosphate (G3P) and malonyl-CoA^9^. The pool sizes of G3P, pyruvate, and AcCoA were higher under salinity stress (Fig. 3). During the lipid accumulation under 2% SS (Day 5 and 7), the pool sizes of G3P, pyruvate, and AcCoA were higher than under 0% SS. When combined the results obtained in Fig. 2, the pool sizes of these metabolites would be corresponding to the lipid content and vital for lipid accumulation.

### Effects of salinity on ^13^C incorporation from CO_2_ into metabolic intermediates

The newly accumulated level of each metabolic intermediate was determined by an *in vivo*^13^C-labeling assay^31^ using ^13^CO_2_. ^13^CO_2_ was transported and then fixed into 3PGA by ribulose 1,5-bisphosphate carboxylase/oxygenase (RuBP). The assimilation rate of carbon into each metabolite is reflected by the ^13^C labeling amounts and thus can help elucidate the dynamics of starch and lipid biosynthesis. ^13^C labeling, in this study, is defined as the amount of carbon that is newly incorporated into metabolites. Figure 4 shows the ^13^C labeling for several metabolites in cells cultivated under 0% and 2% SS. All these^13^C-labeled metabolites were obviously decreased at from Day 3 to Day 7. Higher ^13^C labeling values for F6P, G6P, and glucose 1-phosphate (G1P) were observed under 0% SS than 2% SS on Day 5, whereas the ^13^C labeling of G3P was significantly increased at Day 3 and 5 under 2% SS. Moreover, the ^13^C labeling of pyruvate and AcCoA was higher under 2% SS at Day 5 and 7.

### Relative quantification of mRNA levels related to lipid and starch synthesis with/without salinity stress

**Figure 1:**
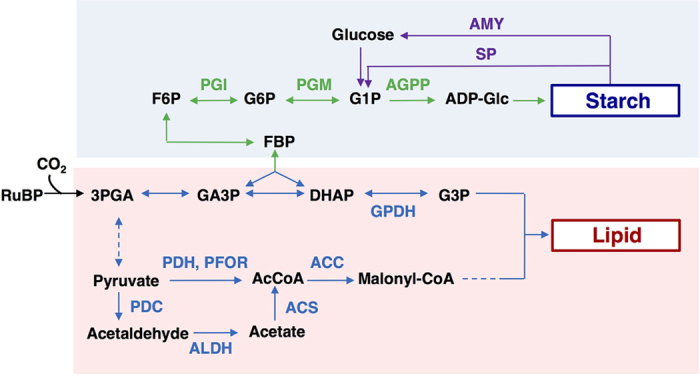
Carbon partitioning from CO_2_ to starch and lipid accumulation and the key enzymes in *Chlamydomonas* spp. Metabolites: ribulose 1,5-bisphosphate carboxylase/oxygenase; RuBP, 3-phosphoglycerate; 3 PGA, glyceraldehyde 3- phosphate; GA3P, fructose 1,6-bisphosphate; FBP, dihydroxyacetone phosphate; DHAP, fructose 6-phosphate; F6P, glucose 6-phosphate; G6P, glucose 1-phosphate; G1P, ADP-Glucose; ADP-Glc, glycerol 3-phosphate; G3P, acetyl-CoA; AcCoA. Enzymes: phosphoglucoisomerase; PGI, phosphoglucomutase; PGM, ADP-Glc phosphorylase; AGPP, α-amylase; AMY, starch phosphorylase; SP, glycerol 3-phosphate dehydrogenase; GPDH, pyruvate dehydrogenase; PDH, pyruvate-ferredoxin oxidoreductase; PFOR, AcCoA carboxylase; ACC, pyruvate decarboxylase; PDC, acetaldehyde dehydrogenase; ALDH, AcCoA synthetase; ACS. Dotted lines represent multiple reaction steps.

A better understanding of salinity-induced starch-to-lipid conversion in JSC4 requires time-course transcription analyses of the genes related to starch biosynthesis/degradation and lipid biosynthesis. Genome sequencing revealed that key genes in the lipid and starch metabolism pathway are conserved between *C. reinhardtii* and JSC4 (Fig. 1). The switching of starch/lipid synthesis induced by salinity stress described above was substantiated by quantifying mRNA levels by quantitative real-time PCR (qPCR) (Fig. 5).

Transcription of genes involved in synthesis and degradation of starch^10^,^11^ was measured simultaneously. At Day 5, the mRNA levels of starch-synthesis-related genes such as *PGI* and *PGM* were significantly higher under 0% SS than under 2% SS. In particular, the level of *AGPP* drastically increased more than 10,000-fold from Day 3 to Day 5. The mRNA levels of enzymes involved in starch degradation, such as *SP1* and *SP2*, were significantly higher under 2% SS than under 0% SS. The levels of *SP1* and *SP2* dramatically increased from Day 3 to Day 5, and the level of *SP2* further increased 13.5-fold from Day 5 to Day 7. The level of *AMY1* under salinity stress was higher than that under 0% SS at Day 3 and 5, while *AMY2* expression was lower under 2% SS.

Next, transcription of genes involved in lipid synthesis^11^,^12^,^13^,^14^,^15^,^16^,^17^ was measured. Determination of the mRNA expression involved in lipid synthesis showed that *PFOR* mRNA under 2% SS increased approximately 10.5-fold from Day 3 to Day 5 and then remained constant; these levels are much higher than under 0% SS. The levels of *PDC, ALDH, ACS2*, and *ACS3* under 2% SS were also significantly higher than under 0% SS. In particular, *PDC* showed 8.3-fold increase from Day 3 to Day 7 under 2% SS. *ACS2* and *ACS3* were enhanced from Day 3 to Day 5 under 2% SS (5.7- and 18.4-fold, respectively). ACC in *Chlamydomonas* spp. contains biotin carboxyl carrier protein (BCCP), biotin carboxylase (BC), and carboxyltransferase α-subunit (αCT) and β-subunit (βCT)^16^. The mRNA levels of *BCCP, BC, αCT*, and *βCT* was constantly higher under 2% SS. *GPDH* expression was higher under 2% SS at Day 3, but dramatically decreased thereafter, whereas extremely low signals of *GPDH* were detected under 0% SS throughout the cultivation period.

### Determination of starch-degrading enzyme activity under salinity stress

AMY and SP activities in the cells were evaluated on Day 5 and 7 to investigate the starch degradation pathway affected by salinity. AMY activity was not largely affected by the addition of SS (Fig. 6). In contrast, SP activity was significantly increased under 2% SS (3.2- and 10.5-fold at Day 5 and 7, respectively).

## Discussion

The economic feasibility of microalgal biodiesel can be enhanced by selecting/engineering a strain capable of maintaining high biomass production and rapid accumulation of lipid under environmental stress^6^. We previously reported that JSC4 is not only a robust producer of both biomass and lipid but a strain with high tolerances of irradiance and salt, which is quite suitable to apply for seawater-based outdoor cultivation^23^,^31^. Nitrogen stress is one of the key factors for enhancing lipid accumulation in many microalgal species^3^, however, the single stress of nitrogen depletion could not always induce the lipid accumulation of microalgae. In some microalgal strains, combining the dual stresses are required^6^. Salinity is also an important factor affecting the lipid content of some microalgae species, but their lipid productivity under high salinity stress is usually lower due to the concomitant strong inhibition of cell growth^6^. Metabolic profiling enables us to discover the metabolic mechanism for improving lipid accumulation in a microalga^34^. By utilizing knowledges of the lipid metabolism pathway, we can construct an oil-overproducing algal strain through genetic engineering^20^,^34^. However, the mechanism of salinity-induced lipid synthesis in JSC4 has not been elucidated in previous studies^23^,^31^,^32^. In this study, dynamic metabolic profiling together with transcription analysis under salinity stress were conducted for the first time, and the switching mechanisms from starch to lipid synthesis in JSC4 was demonstrated. We also successfully identified the metabolic rate-limiting step (*e.g.*, pyruvate-to-AcCoA) and several key genes (*e.g*., *ACC, PDC, ALDH, ACS, PFOR*, and *SP*) for enhancing lipid accumulation in JSC4.

**Figure 2:**
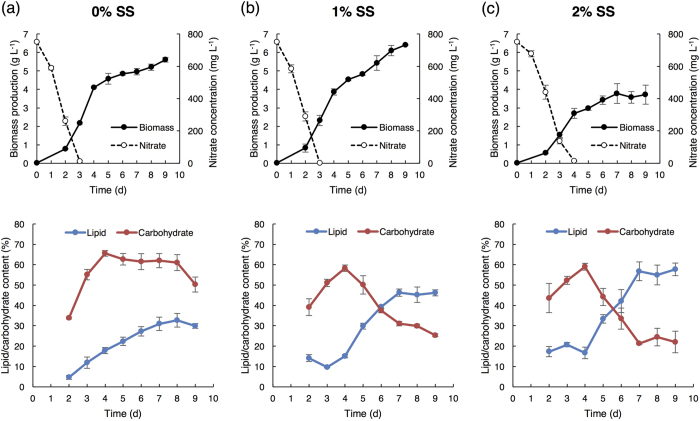
Energy storage compound switched from starch to lipid upon salinity stress. Time-course profiles of biomass production, nitrate concentration, and lipid and carbohydrate content under (**a**) 0%, (**b**) 1%, and (**c**) 2% of sea salt (SS). Error bars indicate the standard deviation (SD) of three replicate experiments.

**Figure 3:**
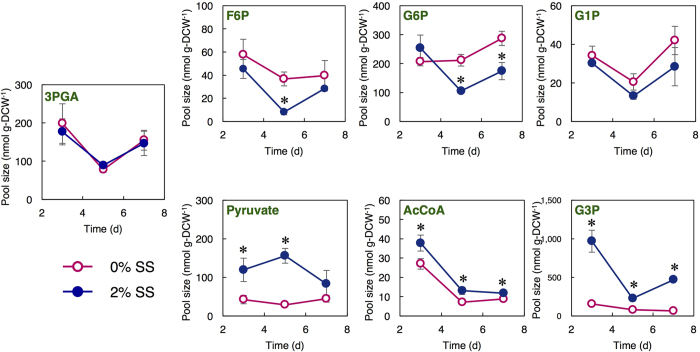
Pool size of metabolic intermediates under different SS concentrations. (0% and 2%) at Day 3, 5, and 7. ○ (pink) and • (blue) indicate 0% and 2% SS, respectively. Error bars indicate the SD of three replicate experiments (*p < 0.05 by Student’s *t*-test).

**Figure 4:**
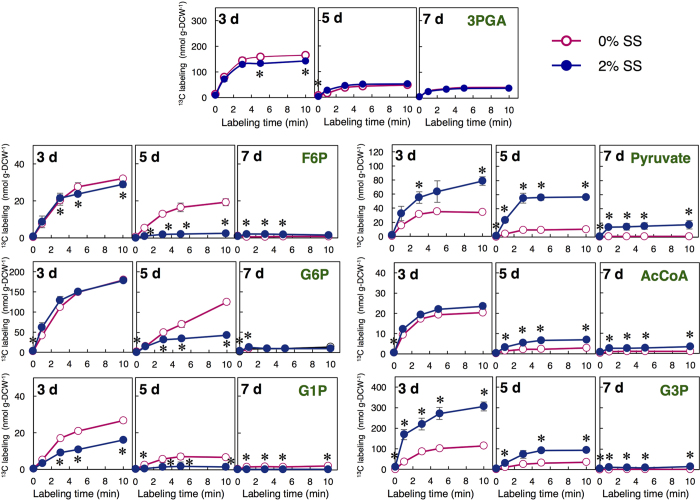
Time course of ^13^C labeling under different SS concentrations (0% and 2%) at Day 3, 5, and 7. ○ (pink) and • (blue) indicate 0% and 2% SS, respectively. Error bars indicate the SD of three replicate experiments (*p < 0.05 by Student’s *t*-test).

We show that salinity stress reduces starch and concomitantly enhances lipid accumulation (Fig. 2), and significantly increases the pool sizes of key lipid-synthesis-related metabolites (Fig. 3). Wang *et al*. indicated that salt causes osmotic stress, resulting in a dramatic accumulation of glycerol in *Dunaliella tertiolecta* and *Dunaliella bardawil*^35^. This is in agreement with the rapid accumulation of G3P under 2% SS shown in this study, since G3P is an important intermediate for glycerol synthesis. In Fig. 4, the ^13^C labeling of G1P was slightly higher at Day 5 under 0% SS, indicating that more G6P would be converted to G1P, and that the reaction from G6P to G1P might be activated to maintain a high starch content. In addition, a much higher ^13^C labeling of G6P was observed at Day 5 under 0% SS. Carbohydrate accumulation in *Chlamydomonas* spp. is mainly regulated by AGPP activity as the rate-limiting step^19^,^21^,^36^. We here propose that enhancing the reaction rate from G6P to G1P could also play an important role in improving starch accumulation in microalgae. In transgenic tobacco plants, overexpression of plastidial PGM increased starch content^37^. This research may support our proposal.

The ^13^C labeling of G3P was significantly higher under 2% SS, particularly at Day 3 and 5 (Fig. 4). The ^13^C labeling of AcCoA under 2% SS was also higher compared to under 0% SS. Thus, the higher ^13^C labeling of G3P and AcCoA under SS may correlate with higher lipid accumulation in the cell. On the other hand, the ^13^C labeling of pyruvate under salinity stress was clearly higher than that of AcCoA, likely due to not only an increase of carbon influx into pyruvate but also a limitation in the conversion to AcCoA. In Fig. 3, pyruvate was accumulated in the presence of SS, while the level of AcCoA was not largely increased. The results suggest that the lipid biosynthesis can be further improved by accelerating the metabolic reaction from pyruvate to AcCoA. By tracking the dynamic carbon flow, we found the likely rate-limiting steps in starch/lipid synthesis (*e.g.*, starch: G6P-to-G1P; lipid: pyruvate-to-AcCoA), and proposed the strategy of starch-to-lipid conversion under salinity stress to effectively enhance lipid accumulation in microalgae. Decreased ^13^C labeling of these metabolites at Day 5 and 7 (Fig. 4) suggests that lipid production was performed not only by using newly incorporated CO_2_ but also by using intracellular carbon sources (*i.e.* carbohydrates).

**Figure 5:**
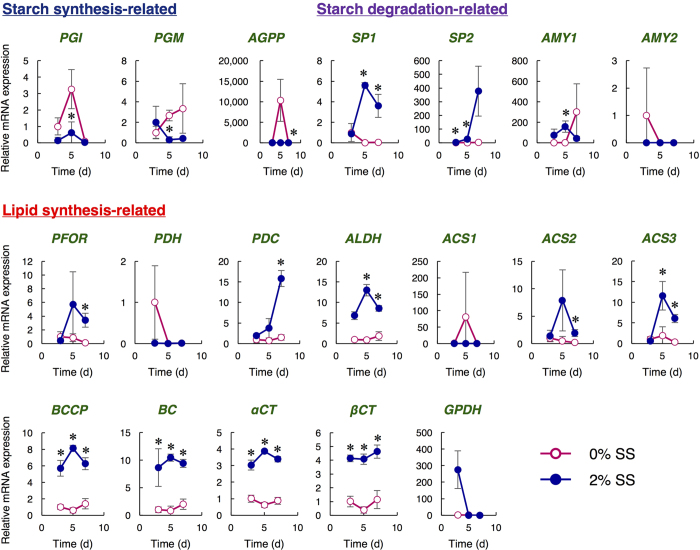
Relative quantification of mRNA under different SS concentrations (0% and 2%) at Day 3, 5, and 7. ○ (pink) and • (blue) indicate 0% and 2% SS, respectively. Biotin carboxyl carrier protein (BCCP), biotin carboxylase (BC), and carboxyltransferase α-subunit (αCT) and β-subunit (βCT) are components of ACC in *Chlamydomonas* sp. Shown are the relative mRNA levels normalized by the level of the *RPL32* and by the level of each gene at the Day 3 under the 0% SS. Error bars indicate the SD of three replicate experiments (*p < 0.05 by Student’s *t*-test).

**Figure 6:**
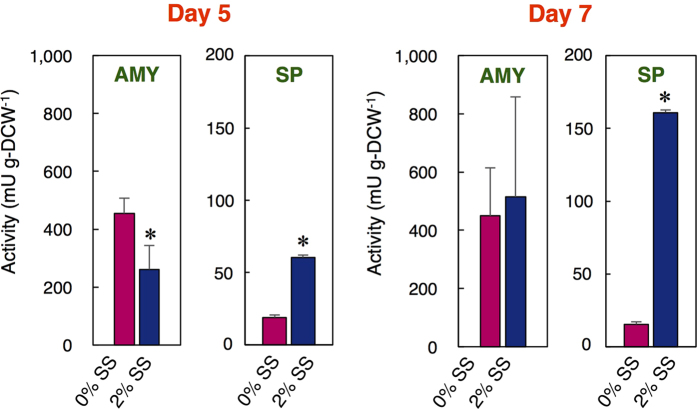
Catalytic activity of AMY and SP at Day 5 and 7. Error bars indicate the SD of three replicate experiments (*p < 0.05 by Student’s *t*-test).

Level of key gene transcripts is important for understanding the metabolic pathway. Although previous transcriptomic analyses in *C. reinhardtii* shed light on carbon partitioning between lipid and starch^9^, the levels of key transcripts related to starch and lipid biosynthesis and starch degradation under salinity stress were here evaluated simultaneously and dynamically for the first time in JSC4 (Fig. 5). Lower levels of the *PGI* and *PGM* transcript under 2% SS indicate that the conversion of F6P to G1P via G6P can be retarded to slow down starch biosynthesis. At Day 5, the drastically increased *AGPP* transcript under 0% SS may correlate to a high content of starch in the cells since the metabolic reaction by AGPP should be the main step for producing starch^21^. Interestingly, the significant enhancement of the *SP1/SP2* transcripts under 2% SS at Day 5 and 7 indicates that *SP1* and *SP2* were immediately activated during salinity stress, thus aiding the switch from starch to lipid synthesis. With the results shown in Fig. 6, these findings clearly indicate that the upregulation of genes related to starch degradation by salinity stress could lead to the production of more SP, and the concomitant generation of more lipids. *C. reinhardtii* possesses functional AMY and SP as starch degradation enzymes^38^, however, it accumulates both lipid and starch under salinity stress^18^. Under salinity stress, the JSC4 cells produce lipid by degrading starch, and this would be the characteristic feature of this strain.

We focused on characterizing the mRNA levels of lipid biosynthesis-related genes (Fig. 5). The dramatic increase in *PFOR* transcript under 2% SS and the extremely low levels of *PDH* transcript was observed. These results raise the possibility that the metabolic reaction from pyruvate to AcCoA may be primarily catalyzed by PFOR, but not PDH. In the oleaginous green alga *Chlorella desiccata*, activation of the PDH-bypass, which consists of PDC, ALDH and ACS, is required for higher lipid biosynthesis under nitrogen deprivation^39^. In JSC4, expression levels of *PDC, ALDH*, and *ACS* were increased under SS. This result suggests that the PDH-bypass might also play important roles in lipid production under salinity condition. Notably, because GPDH is a critical enzyme in catalyzing the reversible conversion of dihydroxyacetone phosphate (DHAP) to G3P, the high level of the *GPDH* transcript at Day 3 under salinity stress indicates that large amounts of G3P would be produced and be available for lipid synthesis, which consistent with the results in Figs 3 and 4. Thus, by using transcription analysis combined with metabolic profiling, we suggest that carbon flow between starch and lipid biosynthesis in microalgae can be regulated by salinity stress. There is a possibility that some gene functions are also regulated at post-translational levels. Further examination of the specific enzymatic activity is required to strengthen the hypothesis obtained in this study.

The pool size of metabolites in *Chlamydomonas* spp. has been evaluated by metabolic profiling analysis. Bölling and Fiehn reported changes in the pool sizes of metabolites in *C. reinhardtii* following the depletion of various components of the growth medium^27^. However, changes in the pool size are not always reflected in the dynamic metabolism^40^. Accordingly, the dynamic metabolic turnover and carbon fraction of these metabolites must be investigated^41^,^42^. Recently, Kempa *et al*. reported the turnover of metabolites in *C. reinhardtii* by analyzing ^13^C isotopomer flow, however, it does not shed light on how to enhance a specific metabolic reaction^41^. Our study is the first report to provide comprehensive information regarding dynamic carbon flux and transcription related to the switching mechanisms between starch and lipid biosynthesis.

Increase in cellular biomass was observed after nitrate depletion (Fig. 2), likely due to the utilization of intracellular nitrogen source including protein. As previously reported, the JSC4 strain demonstrates protein reduction after nitrate depletion^23^. Some algae have shown cellular biomass increase along with intracellular protein reduction after the nitrogen depletion^23^,^28^. Further study is required to elucidate the biomass increment mechanism.

We suppose that the reason why JSC4 accumulates lipid under the salinity condition would be related to the fact that they originally live in the brackish-water region^23^. In the brackish water, nutrients required for photosynthesis might be sufficiently supplied from river. When JSC4 is carried to the open ocean by oceanic current, they might suffer from both starvation and salinity stress, which triggers accumulation of lipid as an energy source for long term survival. As the freshwater microalga *C. reinhardtii*, which was originally isolated from Amherst in the United States^42^, does not carry out the salinity-induced carbon flow switching^18^, it might be a brackish water-specific response to starvation.

## Methods

### Microorganism and growth conditions

*Chlamydomonas* sp. JSC4^23^, isolated from a coastal area of southern Taiwan, was cultivated under phototrophic condition in Modified Bold (MB) 6N medium consisting of 8.8 mM NaNO_3_, 0.22 mM K_2_HPO_4_, 0.3 mM MgSO_4_, 0.17 mM CaCl_2_, 0.43 mM KH_2_PO_4_, 0.43 mM NaCl, and different concentrations of SS (Sigma-Aldrich Co., St. Louis, MO, USA). The levels of metals in the medium are described in the previous report^43^. After 3 days’ pre-culture, cells were inoculated into double-deck photobioreactor^44^, which has a first stage containing 2M NaHCO_3_/Na_2_CO_3_ to supply the desired concentration of CO_2_, and a second stage containing the culture broth, at an initial cell concentration of 20 mg L^−1^. The cells were cultured under the conditions as follows; light intensity, 250 μmol photons m^−2^ s^−1^ (white fluorescent lamps); CO_2_ aeration, 2% CO_2_; temperature, 30 °C.

### Measurement of residual nitrate content

Nitrate concentration was measured using an optical method as previously reported^45^. The broth was centrifuged at 5,000 × *g* for 1 min, and the absorbance of the supernatant diluted 20-fold with distilled water was measured at 220 nm (*i.e*., Abs_220_) using UVmini-1240 UV-VIS spectrophotometer (Shimadzu, Kyoto, Japan). The residual nitrate content was evaluated using an appropriate calibration curve^23^.

### Evaluation of lipid content

Cultivation of JSC4 was carried out under the same conditions as described above. Cells were collected by centrifugation at 5,000 × *g* for 1 min, washed with distilled water twice, and lyophilized. The dried cells were fractured with 0.5 mm glass beads using a multi-bead shocker (Yasui Kikai, Osaka, Japan) at 4 °C. The total lipids were extracted using the mixture of chloroform, methanol and water, and were esterified by Fatty Acid Methylation Kit (Nacalai Tesque, Kyoto, Japan) according to the previous method^46^. The fatty acid methyl esters (FAMEs) were identified and quantified by gas chromatography-mass spectrometry (GC-MS) on a GCMS-QP2010 Plus (Shimadzu) as described previously^23^. Samples were injected onto a DB-23 capillary column (60 m, 0.25 mm internal diameter, 0.15 μm film thickness; Agilent Technologies, Palo Alto, CA, USA). Helium was used as the carrier gas at a flow rate of 2.3 mL min^−1^. The injector, ion source, and interface source temperatures were set at 230, 230, and 250 °C, respectively. The oven temperature was initially set at 50 °C for 1 min, increased from 50 to 175 °C at a rate of 25 °C/min, increased from 175 to 230 °C at a rate of 4 °C/min, and held at 230 °C for 5 min. Supelco 37 Component FAME Mix (Sigma-Aldrich Co.) was utilized as a quantitative standard, and heptadecanoic acid (Sigma-Aldrich Co.) was used as an internal standard. Lipid productivity is calculated as overall lipid produced from Day 0.

### Evaluation of carbohydrate content

The total carbohydrate content in the dried cells, prepared as above, was evaluated using a colorimetric method with an anthrone reagent^47^. The dried cells were incubated in anthrone solution (0.2% (*w*/*v*) anthrone in 75% (*v*/*v*) sulfuric acid) for 15 min at 100 °C. After cooling on ice, an aliquot was centrifuged at 5,000 × *g* for 1 min and the supernatant was measured at 620 nm (*i.e.*, Abs_620_). The carbohydrate content was quantified using a standard curve generated using glucose.

### Metabolic profiling

Cultivation of JSC4 was carried out under the same conditions as described above. Cell sampling was performed according to our previously reported method^23^. Cells were collected on 1-μm pore size polytetrafluoroethylene filter disks (Omnipore; Millipore, Billerica, MA, USA), washed with pre-chilled (4 °C) 20 mM ammonium carbonate, and immediately placed into 1 mL of pre-chilled (−30 °C) methanol containing 12.4 μM piperazine-1,4-bis(2-ethanesulfonic acid) as the internal standard. 300 μL of pre-chilled (4 °C) chloroform and 100 μL of pre-chilled (4 °C) water were added to provide a 10:3:1 (*v*/*v*/*v*) methanol:chloroform:water mixture. The cells were completely fractured with 300 μL of 0.5 mm glass beads using a multi-beads shocker (Yasui Kikai), then 396 μL of distilled water was added. The water layer was filtered through a Millipore 5 kDa cut-off filter and dried under vacuum using a FreeZone 2.5 Plus freeze dry system (Labconco, Kansas City, MO, USA).

The intermediate metabolites in lipid/starch synthesis pathway (*e.g.*, 3 PGA, pyruvate, AcCoA, G3P, F6P, G6P, and G1P) were targeted and determined according to a previously described method^28^. Dried metabolites were dissolved in 20 μL of Milli-Q water and analyzed using a capillary electrophoresis-mass spectrometry (CE-MS) system comprising an Agilent G7100 CE system, an Agilent G6224AA LC/MSD time-of-flight system, and an Agilent 1200 series isocratic HPLC pump equipped with a 1:100 splitter for delivery of the sheath liquid. The CE separations were performed in a fused silica capillary (1 m × 50 μm i.d.) filled with 50 mM ammonium acetate (pH 9.0) for anionic metabolite analyses. The flow rate of the sheath liquid was set at 8 μL min^−1^. The electrospray ionization-mass spectrometry analyses were conducted in negative ion mode. Mass data were acquired at a rate of 1 spectra s^−1^ over the mass-to-charge ratio (*m/z*) range 70–1000.

### *In vivo*^13^C-labeling

Using the same cell cultures as the metabolic profiling, *in vivo*^13^C-labeling was performed using sodium ^13^C-bicarbonate (NaH^13^CO_3_) as a carbon source as described previously^31^. Cells were harvested from culture broth at Day 3, 5, and 7 and resuspended in labeling medium (25 mM NaH^13^CO_3_) at the same cell density as in the culture broth. After time-course labeling for 1–10 min to monitor the accumulation rates, metabolites in approximately 10 mg of wet cells were analyzed using CE-MS as described above. The ratio of ^13^C to total carbon in each metabolite was calculated by searching for mass shifts between the ^12^C and ^13^C mass spectra^31^. ^13^C labeling, defined as “pool size (nmol g-DCW^−1^) × ^13^C fraction (%) (Fig. S2”), was used to evaluate the quantitative metabolic flux in the cells.

### Transcript analysis

Cultivation of JSC4 was performed under the same conditions as described above. Cells were harvested from the broth by centrifugation at 5,000 × *g* for 1 min, frozen in liquid nitrogen, and milled using a mortar^48^. Total RNA was isolated from the frozen cell powder using an RNeasy Plus Universal Kit (QIAGEN, Tokyo, Japan). For qPCR experiments, complementary DNA was synthesized from approximately 100 ng of total RNA using a ReverTra Ace qPCR RT Master Mix with gDNA Remover (TOYOBO, Osaka, Japan). qPCR was performed with THUNDERBIRD SYBR qPCR Mix (TOYOBO) using Mx qPCR Systems (Agilent). The average threshold cycle values were evaluated throughout the logarithmic amplification phase using triplicate samples, and were normalized by the level of the *RPL32* (encoding ribosomal protein large subunit) and by the level of each gene at the Day 3 under 0% SS to evaluate relative levels of RNA transcription. The qPCR primers (Table S2) were designed based on each predicted gene sequence from the genome sequence, which was determined by GS FLX + (Roche) and MiSeq (Illumina) systems using genome DNA extracted by DNeasy Plant mini kit (QIAGEN) from the JSC4 cells.

### Enzyme activity analysis

Cultivation of JSC4 was performed under the same conditions as described above. AMY and SP activities were analyzed from approximately 100 mg and 60 mg of wet cells, respectively. After harvesting by centrifugation at 5,000 × *g* for 1 min, cells were suspended in 1 mL of 50 mM Hepes-NaOH buffer (pH 7.0) containing 2 mM EDTA and 2 mM CaCl_2_, then were frozen and thawed with liquid nitrogen and 30 °C water four times^38^. The cells were broken by sonicating on ice for 30 s of 50% pulses; 30 s cooling period, five cycles. The suspension was centrifuged at 20,000 × *g* for 20 min at 4 °C, and the supernatant was purified on a PD-10 column system (GE-Healthcare Bio-Sciences KK, Tokyo, Japan). AMY activity of the cell extract was analyzed using an α-Amylase assay kit (Kikkoman Biochemifa Company, Tokyo, Japan)^49^. Hydrolysis of a model substrate, 2-chloro-4-nitrophenyl 6^5^-azido-6^5^-deoxy-β-maltopentaoside was detected by 2-chloro-4-nitrophenol (CNP) generation. 1 unit of the enzyme activity was defined as the amount of enzyme required to liberate 1 μmol of CNP per minute at 37 °C.

SP activity of the cell extract was evaluated according to a previous report^50^ with some minor modification. 50 μL of purified protein suspension was added to 395 μL of 50 mM HEPES-NaOH buffer (pH 7.0) containing 10 mM inorganic phosphoric acid and 10 mg mL^−1^ soluble starch (CAS number: 9005-84-9, Nacalai Tesque), incubated at 30 °C for 1 h, and boiled for 5 min to stop the catalytic reaction. Then 500 μL of 50 mM Tris-HCl buffer (pH 7.0) containing 120 mM MgCl_2_, 0.05 mM glucose 1,6-diphosphate, and 0.5 mM NADP was mixed with the suspension, 4 units of PGM and 2 units of G6P dehydrogenase were added, and reacted for 30 min at room temperature. The production of NADPH was monitored by absorbance at 365 nm using UVmini-1240 (Shimadzu). 1 unit of the enzyme activity was defined as the amount of enzyme required to liberate 1 μmol of G1P per minute at 30 °C.

## Additional Information

**How to cite this article:** Ho, S.-H. *et al*. Dynamic metabolic profiling together with transcription analysis reveals salinity-induced starch-to-lipid biosynthesis in alga *Chlamydomonas* sp. JSC4. *Sci. Rep.* **7**, 45471; doi: 10.1038/srep45471 (2017).

**Publisher's note:** Springer Nature remains neutral with regard to jurisdictional claims in published maps and institutional affiliations.

## Supplementary Material

Supplementary Information

## Figures and Tables

**Table 1:** Time-course profiles of biomass productivity under different SS conditions (0%, 1%, and 2%).

SS (%)	Cultivation time (d)	Biomass productivity (mg-DCW L^−1^ d^−1^)	Lipid content (%)	Lipid productivity (mg L^−1^ d^−1^)
0	3	724.3 ± 16.5	11.9 ± 2.8	86.2 ± 7.0
	5	915.0 ± 58.3	22.4 ± 2.0	205.0 ± 5.8
	7	708.4 ± 24.6	31.0 ± 3.3	219.4 ± 22.4
1	3	772.2 ± 92.0	9.7 ± 0.2	74.6 ± 5.3
	5	907.3 ± 13.0	29.8 ± 1.4	270.7 ± 10.8
	7	775.2 ± 56.3	46.5 ± 1.8	358.9 ± 20.6
2	3	510.3 ± 45.6	20.8 ± 1.0	105.9 ± 12.9
	5	569.5 ± 37.9	33.4 ± 2.1	198.2 ± 20.1
	7	539.3 ± 76.6	56.9 ± 4.6	306.1 ± 19.2

Values are the averages of three replicated experiments, ±SD.
