# Supplementary material for: Dynamic metabolic profiling together with transcription analysis reveals salinity-induced starch-to-lipid biosynthesis in alga Chlamydomonas sp. JSC4
Source: Sci Rep. 2017 Apr 4;7:45471. doi: 10.1038/srep45471 (PMC5379629; doi:10.1038/srep45471)
Supplement: Supplementary Information [file srep45471-s1.pdf]

Dynamic metabolic profiling together with transcription analysis reveals salinity-induced starch-to-lipid biosynthesis in alga *Chlamydomonas* sp. JSC4

Shih-Hsin Ho, Akihito Nakanishi, Yuichi Kato, Hiroaki Yamasaki, Jo-Shu Chang, Naomi Misawa, Yuu Hirose, Jun Minagawa, Tomohisa Hasunuma*, Akihiko Kondo.

Supplementary Figure S1. TEM micrographs of JSC4 cultivated under 0% and 2% SS.

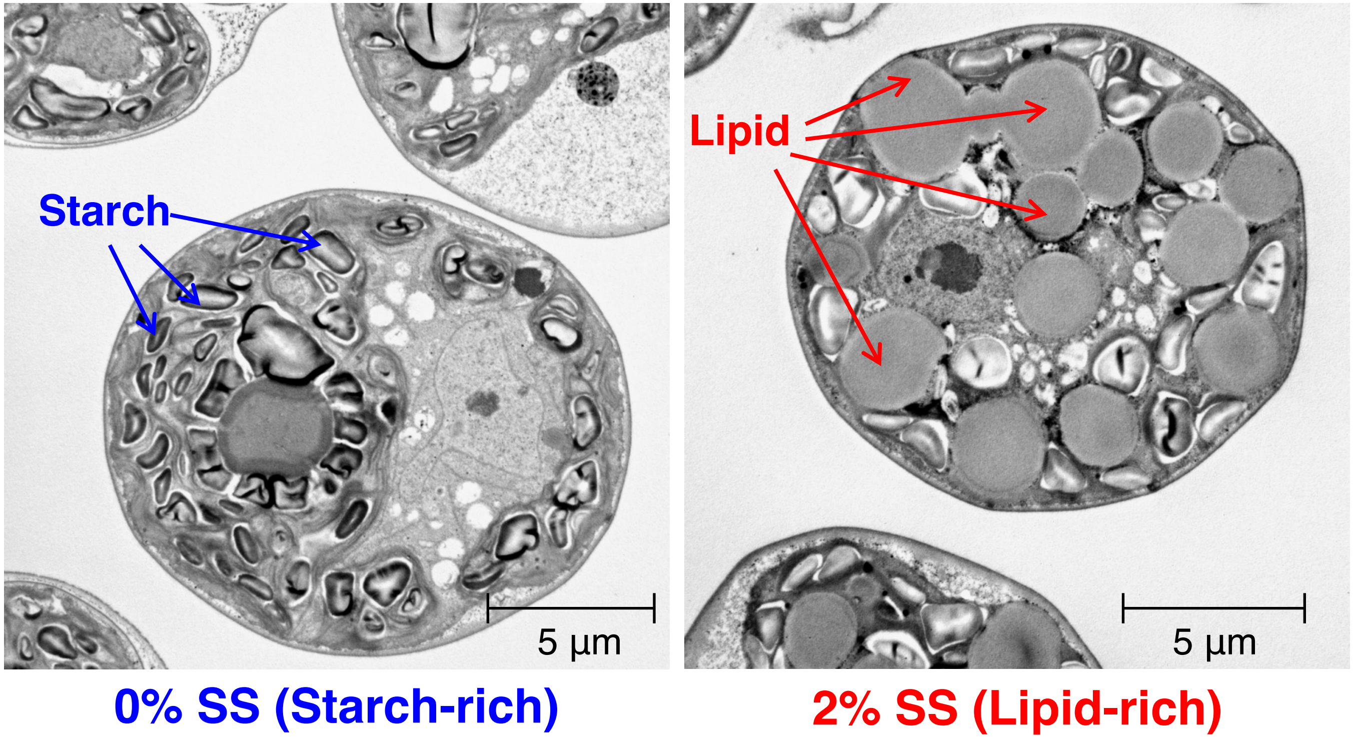

Supplementary Figure S2. Time course of ^{13}C fraction under different SS concentrations (0% and 2%) at Days 3, 5, and 7.

○ (pink) and ● (blue) indicate 0% SS and 2% SS, respectively. Error bars indicate the SD of three replicate experiments (*: $p < 0.05$ by Student's t-test).

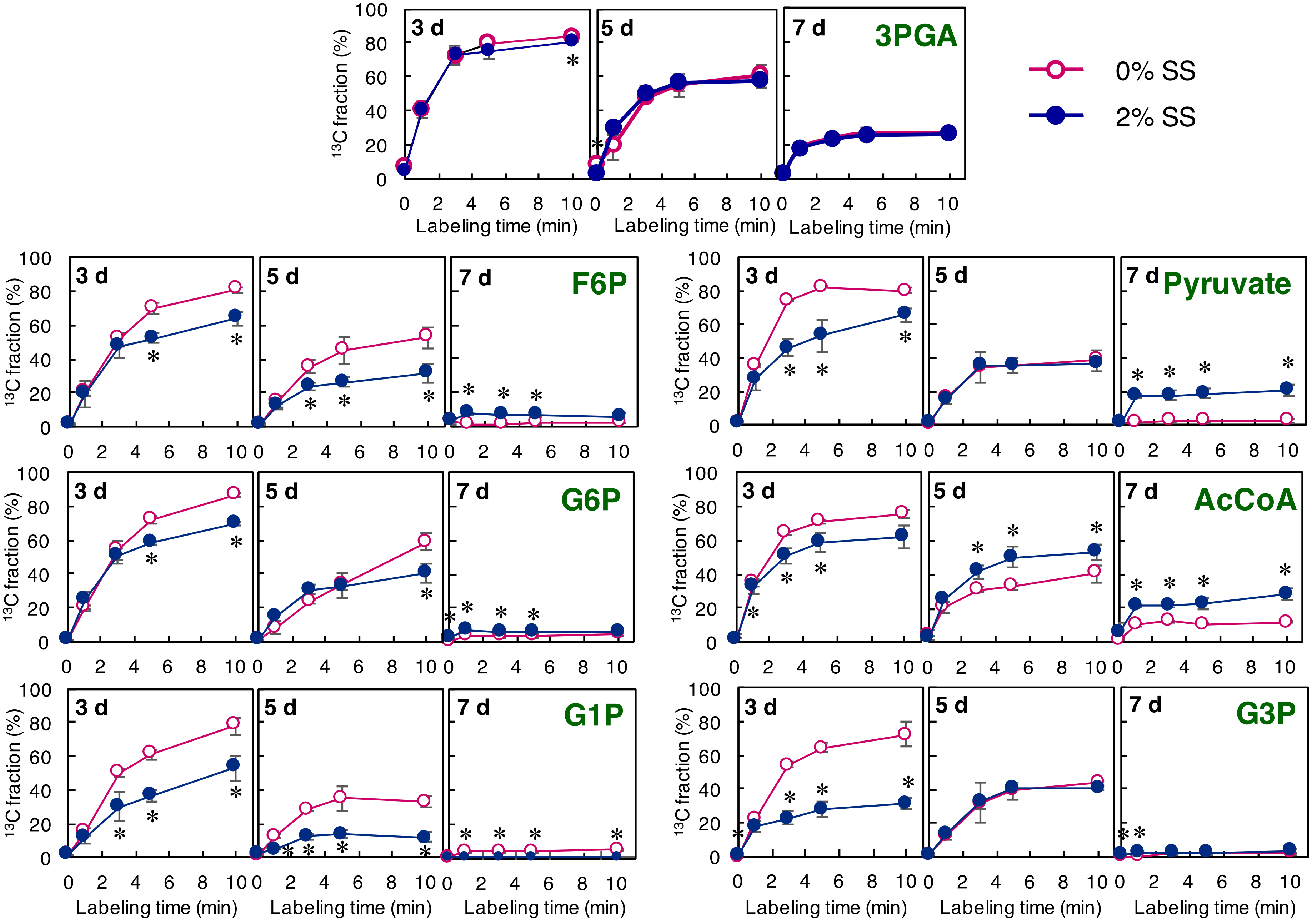

Supplementary Table S1. Comparison of lipid productivity of *Chlamydomonas* sp. JSC4 with those of other green algae, as reported in the literature.

Strain	Biomass productivity ($mg\ L^{-1}\ d^{-1}$)	Lipid productivity ($mg\ L^{-1}\ d^{-1}$)	Reference
Nannochloropsis sp. F&M-M24	180	55	1
Dunaliella tertiolecta ATCC30929	100	70	2
Isochrysis zhangjiangensis	345	141	3
Nannochloropsis oculata NCTU-3	480	142	4
Chlorella vulgaris	358	167	5
Chlorella sorokiniana	246	189	6
Chlamydomonas sp. JSC4 (1% SS)	775	358	This study

1. Rodolfi, L. *et al*. Microalgae for oil: Strain selection, induction of lipid synthesis and outdoor mass cultivation in a low-cost photobioreactor. *Biotechnol. Bioeng.* **102**, 100-112 (2009).
2. Takagi, M., Karseno & Yoshida, T. Effect of salt concentration on intracellular accumulation of lipids and triacylglyceride in marine microalgae *Dunaliella* cells. J. *Biosci. Bioeng*. **101**, 223-226 (2006).
3. Feng, D., Chen, Z., Xue, S. & Zhang, W. Increased lipid production of the marine oleaginous microalgae *Isochrysis zhangjiangensis* (Chrysophyta) by nitrogen supplement. *Bioresour. Technol*. **102**, 6710-6716 (2011).
4. Chiu, S. Y. *et al*. Lipid accumulation and CO_2 utilization of *Nannochloropsis oculata* in response to CO_2 aeration. *Bioresour. Technol*. **100**, 833-838 (2009).
5. Chen, W. *et al*. A high throughput Nile red method for quantitative measurement of neutral lipids in microalgae. *J. Microbiol. Methods* **77**, 41-47 (2009).
6. Chen, C. Y. *et al*. Enhancing microalgal oil/lipid production from *Chlorella sorokiniana* CY1 using deep-sea water supplemented cultivation medium. *Biochem. Eng. J*. **77**, 74-81 (2013).

Supplementary Table S2. Sequences of primers used for qPCR.

Primer name	Definition	Primer sequence (5' to 3')
RPL32 F	Ribosomal protein L32	acaagaagactcgccacacg
RPL32 R		cgttgatgacgcagatgttg
PGI F	Phosphoglucose isomerase	cacgaccatacaccccaatc
PGI R		gccttgttccacaccatcc
PGM F	Phosphoglucomutase	tcagccgctacgactacgag
PGM R		cggacaggcggaaaatg
AGPP F	ADP-glucose phosphorylase	aactccacctcgctcaacc
AGPP R		ctcgatggccctgttcttg
SP1 F	Starch phosphorylase 1	tctacttcctgcccgactacaac
SP1 R		gaacacgaacacctcctccac
SP2 F	Starch phosphorylase 2	aacacccaccacctcaacaac
SP2 R		gatttcatgcaccaagccatac
AMY1 F	α-Amylase 1	tcagcgtcggaatcaaacc
AMY1 R		taaacacctccgtccatctcatc
AMY2 F	α-Amylase 2	catcaagatcaacggcaagg
AMY2 R		cgcaacagcacatacacagg
PFOR F	Pyruvate ferredoxin oxidoreductase	cgtggcggtgtttgaga
PFOR R		ggtgttgctggcgatga
PDH F	Pyruvate dehydrogenase	acctgccccttatcgtcatc
PDH R		gtggttgcgtcttgcgtatc
PDC F	Pyruvate decarboxylase	acgcgtatggggtaaagatacg
PDC R		atagcgcgagaaaaccgttg
ALDH F	Aldehyde dehydrogenase	tcgtgcacgagtctgtatacg
ALDH R		ttgttgaactggtcgctgtc
ACS1 F	AcCoA synthetase 1	caccctgcctcaatagtgtcc
ACS1 R		atggctcggcttcttctcc
ACS2 F	AcCoA synthetase 2	cccgtcaacatccagttcttc
ACS2 R		tctggcggtatgtgattgtg
ACS3 F	AcCoA synthetase 3	ctggagcaagcccatatacaac
ACS3 R		ccctcccagtagaacgcaac
GPDH F	Glycerol 3-phosphate dehydrogenase	aacacgctgcacgaaaacac
GPDH R		ttgctgacgcagatgatgg
BCCP F	Biotin carboxyl carrier protein	aaatgcagctcaacctgacg
BCCP R		cgtcgaacgccatcatgttc
BC F	Biotin carboxylase	tggagatgaacacccgcatc
BC R		acacgatgtcctcctgcttg
αCT F	carboxyl transferase α subunit	aacctcatcatgcagaacgc
αCT R		tccatgacgccgaacttgac
βCT F	carboxyl transferase β subunit	caagatggccaaggctgttg
βCT F		ttgcagccaaagcagatgtg
